# First‐Principles Design of Cubic Spinel ZnIn_2_S_4_ With Enhanced Carrier Transport, Optical Response, and Mechanical Stability for Photocatalytic and Optoelectronic Applications

**DOI:** 10.1002/open.70325

**Published:** 2026-10-28

**Authors:** M. Rahman, N. Y. Tanisa, M. M. Roshid, Y. Yildirim, M. S. A. Khan

**Affiliations:** ^1^ Department of Physics Uttara University Dhaka Bangladesh; ^2^ Department of Physics Begum Rokeya University Rangpur Bangladesh; ^3^ Department of Mathematics Hamdard University Bangladesh Munshiganj Bangladesh; ^4^ Department of Computer Engineering Biruni University Istanbul Turkey; ^5^ Mathematics Research Center Near East University Nicosia Cyprus Turkey; ^6^ Department of Electrical & Electronic Engineering Presidency University Dhaka Bangladesh

**Keywords:** carrier transport, density functional theory (DFT), electronic structure, optoelectronic applications, ZnIn_2_S_4_

## Abstract

ZnIn_2_S_4_ is a ternary sulfide semiconductor that has attracted significant interest because of its promising photoelectric and photocatalytic properties, which are strongly governed by its electronic structure and carrier transport behavior. In this study, first‐principles density functional theory (DFT) calculations are performed to investigate the structural, electronic, carrier transport, optical, mechanical, and dynamical properties of cubic spinel ZnIn_2_S_4_. The optimized lattice parameter is calculated as 7.555 Å, showing excellent agreement with the reported value with only 0.605 % deviation. The electronic structure reveals an indirect semiconducting nature with bandgaps of 1.289 and 1.123 eV obtained from spin‐polarized and nonspin‐polarized GGA‐PBE calculations, respectively. The calculated electron and hole effective masses are 0.129 and 1.970*m*_e_, leading to estimated mobilities of 136.3 and 8.91 cm^2 ^V^−1 ^s^−1^, respectively. Optical calculations demonstrate strong visible‐to‐ultraviolet light interaction with a static dielectric constant of 7.66, maximum refractive index of 3.34, and enhanced absorption response. Mechanical properties confirm structural robustness with a Hill bulk modulus of 75.896 GPa, Young’s modulus of 98.535 GPa, and Poisson’s ratio of 0.28362. Phonon dispersion analysis confirms dynamical stability through the absence of imaginary modes. These findings highlight cubic ZnIn_2_S_4_ as a promising semiconductor for light‐responsive and optoelectronic applications.

## Graphical abstract

First‐principles calculations reveal that cubic spinel ZnIn_2_S_4_ exhibits stable structural characteristics, an indirect bandgap, efficient electron transport, strong visible‐light optical response, and robust mechanical behavior. The combined electronic, optical, carrier transport, and stability features establish ZnIn_2_S_4_ as a promising semiconductor platform for next‐generation light‐responsive and optoelectronic applications.

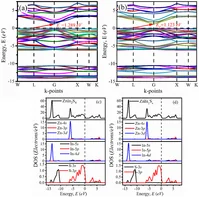

## 1 Introduction

Photocatalysis is widely recognized as an ideal energy conversion technology, enabling the direct conversion of solar energy into clean hydrogen through water splitting [1, 2]. Despite its promise, most photocatalysts still face challenges such as low solar utilization, limited carrier density, and rapid electron–hole recombination, which hinder practical efficiency [3, 4]. Thus, optimizing photocatalysts remains crucial for advancing solar‐to‐hydrogen conversion. Semiconductor‐based photocatalysis, driven by renewable solar energy, is considered a promising solution. The pioneering work of Fujishima and Honda in 1972 demonstrated hydrogen evolution via photoelectrochemical water splitting with TiO_2_ under ultraviolet light [5], inspiring extensive research into semiconductor photocatalysts for water splitting [6] and related applications such as CO_2_ reduction [3], pollutant removal, bacterial disinfection, and selective organic synthesis [7, 8].

Among available photocatalysts, TiO_2_ remains the most extensively studied [5]. However, its wide bandgap (~3.2 eV) restricts absorption to ultraviolet light, resulting in low solar energy utilization [9]. To address this limitation, research has progressed along two main directions: (i) modifying TiO_2_ through doping, oxygen vacancies, surface engineering, and nanotechnology to extend activity into the visible range [10, 11]; and (ii) exploring new material systems with superior photocatalytic properties and visible‐light responses [12, 13]. Metal chalcogenides such as CdS and ZnS have been investigated, but issues of photocorrosion and wide bandgaps hinder their practical application [14, 15]. Recent studies have demonstrated that structural modification, compositional tuning, and theoretical analysis are effective strategies for tailoring the intrinsic properties of functional materials. Element substitution and interaction approaches can modify material stability and performance for energy‐related applications [16], while understanding dynamic responses and material behavior through theoretical modeling provides valuable insights into advanced functional systems [17]. In addition, spinel‐based materials exhibit tunable structural, electronic, dielectric, and functional properties, making them attractive for electronic and optoelectronic applications [18].

Recent advances in photocatalysis have moved beyond the traditional TiO_2_ framework, leading to the development of a wide range of visible‐light responsive materials with tailored structures and properties. TiO_2_ derivatives such as TiO_2__−*x*_N*_x_* [19] and MIL‐125 (Ti) [20] extend TiO_2_ activity into the visible spectrum through doping and hybrid frameworks, while simple oxides like Fe_2_O_3_ [21] and WO_3_ [22] provide earth‐abundant alternatives with strong absorption. Complex oxides, including Ag_3_PO_4_ [23], Bi_2_MoO_6_ [24], and Bi_2_WO_6_ [25], exhibit unique band structures and high oxidative power, offering new pathways for efficient charge separation. Beyond oxides, metal chalcogenides such as CdS [26] deliver narrower bandgaps and enhanced visible‐light activity, though stability remains a challenge. Meanwhile, graphitic carbon nitride (g‐C_3_N_4_) [27, 28] has emerged as a versatile, metal‐free photocatalyst with tunable electronic properties, suitable for hydrogen evolution, CO_2_ reduction, and pollutant degradation. Collectively, these materials demonstrate the dynamic effort to design photocatalysts that efficiently harness visible light, thereby advancing solar hydrogen production and contributing to sustainable energy and environmental development.

Zinc thioindate (ZnIn_2_S_4_), a ternary AB_2_X_4_ chalcogenide, has attracted considerable attention as a visible‐light photocatalyst owing to its high optical utilization, low dark resistance, and favorable optoelectronic properties. It exists in cubic, rhombohedral, and hexagonal polymorphs, with the thermodynamically stable hexagonal phase (P6_3_mc, No. 186) exhibiting enhanced catalytic activity, particularly for cationic dye degradation [13]. Although the hexagonal phase has been extensively studied, the cubic spinel phase of ZnIn_2_S_4_ remains comparatively less explored despite its distinct three‐dimensional crystal framework. The unique arrangement of Zn and In cations within the spinel lattice can significantly influence its electronic structure, carrier transport, optical response, and mechanical stability, making it an important candidate for fundamental investigations and potential semiconductor applications. Chai et al. demonstrated that Pt‐loaded hexagonal ZnIn_2_S_4_ achieved a hydrogen evolution rate of 842 μmol h^−1^ and an apparent quantum efficiency of 34.3% [29], underscoring its potential for solar hydrogen production. As a direct‐bandgap semiconductor (2.06–2.85 eV), ZnIn_2_S_4_ responds to visible light up to ~570 nm [30], making it more efficient than wide‐bandgap sulfides such as ZnS (3.7 eV) [8, 28]. Compared with conventional sulfides like CdS [26] and Sb_2_S_3_ [31], ZnIn_2_S_4_ is less toxic yet displays comparable optical properties, while being relatively facile to synthesize due to abundant raw materials and simple composition. Moreover, it demonstrates considerable chemical stability during photocatalysis [8].

These combined advantages—low toxicity, tunable bandgap, ease of fabrication, and structural versatility—position ZnIn_2_S_4_ as an energy and optoelectronic applications. Beyond hydrogen evolution, ZnIn_2_S_4_ has been actively investigated next‐generation photocatalyst for sustainable CO_2_ reduction [3, 8, 13], selective organic synthesis, and environmental remediation [32]. Its ability to harness visible light efficiently, coupled with chemical robustness, makes it a superior alternative to traditional sulfides. Collectively, these findings highlight ZnIn_2_S_4_ as a highly promising material system for advancing solar energy conversion and environmental sustainability, bridging fundamental research with practical applications in photocatalysis.

Recent studies on advanced optoelectronic materials highlight the importance of efficient light absorption, charge transport, stability, and device integration for next‐generation semiconductor technologies [33, 34]. Similarly, the calculated properties of cubic ZnIn_2_S_4_ suggest potential for photodetectors, photovoltaic‐related systems, and other optoelectronic applications. However, practical implementation requires addressing challenges such as defect control, carrier recombination, and device optimization.

Despite its advantages, ZnIn_2_S_4_ still faces several challenges, including rapid electron–hole recombination, limited charge transport, and structural defects, which can reduce carrier lifetime and photocatalytic efficiency. Since photocatalytic processes involve photon absorption, charge generation/separation, and surface reactions [35], the overall performance of ZnIn_2_S_4_ is strongly influenced by its electronic structure, optical response, crystal framework, and surface characteristics [36]. To improve its functional properties, various modification approaches have been investigated, including morphology regulation, elemental doping, heterojunction construction, and cocatalyst integration. For example, morphology control using different ZnIn_2_S_4_ architectures can influence photocatalytic activity [37], while elemental doping such as Cu incorporation can modify the electronic structure and enhance visible‐light absorption [38]. Heterojunction construction, exemplified by ZnIn_2_S_4_/K_2_La_2_Ti_3_O_10_ composites, improves charge separation and hydrogen evolution [39]. Similarly, cocatalyst loading and hybridization with carbon dots have yielded superior pollutant degradation performance [40]. These advancements demonstrate the importance of understanding the intrinsic structural, electronic, optical, and carrier transport properties of ZnIn_2_S_4_ through first‐principles investigations. Therefore, optimizing these fundamental properties is essential for developing ZnIn_2_S_4_‐based materials for future energy conversion and environmental applications.

## 2 Research Methodology

First‐principles calculations based on density functional theory (DFT) were employed to investigate the structural, mechanical, effective mass, and optoelectronic properties of cubic spinel ZnIn_2_S_4_. DFT provides a reliable theoretical framework for predicting the ground‐state properties of condensed matter systems by solving the Kohn–Sham equations and has been extensively applied for the exploration of semiconductor materials [41]. In this study, all calculations were performed using the CASTEP (Cambridge Serial Total Energy Package) module incorporated in Materials Studio, which utilizes a plane‐wave pseudopotential approach for electronic structure calculations [42]. This computational methodology has been widely employed for investigating the structural, electronic, and optical properties of advanced functional materials [43, 44]. The geometry optimization and subsequent physical property calculations were carried out using both spin‐ and nonspin‐polarized DFT approaches with the generalized gradient approximation (GGA) parameterized by Perdew–Burke–Ernzerhof (PBE) for the exchange–correlation functional [45]. The electron–ion interactions were described using ultrasoft pseudopotentials, enabling an accurate treatment of the valence electronic states with reduced computational requirements. This approach provides a reliable framework for evaluating the intrinsic structural, electronic, mechanical, and optical characteristics of cubic ZnIn_2_S_4_.

The cubic spinel ZnIn_2_S_4_ structure was constructed based on the experimentally reported $Fd\bar{3}m$ (No. 227) space group [46]. The structural relaxation was performed by allowing the lattice parameters and atomic coordinates to fully optimize without imposing additional constraints until the minimum‐energy configuration was achieved. The valence electron configurations used in the calculations were Zn (3*d* ^10^4*s* ^2^), In (4*d* ^10^5*s* ^2^5*p* ^1^), and S (3*s* ^2^3*p* ^4^), ensuring an appropriate description of the electronic states responsible for the bonding characteristics and optoelectronic response of the compound.

In the present study, the primitive P1 cell was employed for structural optimization to allow complete relaxation of atomic positions without imposing additional symmetry constraints. Although the ideal cubic spinel ZnIn_2_S_4_ structure belongs to the $Fd\bar{3}m$ space group, the use of a P1 representation does not alter the fundamental crystal framework; it only removes symmetry restrictions during optimization. The relaxed structure maintains the characteristic cubic spinel arrangement, and the calculated properties represent the equilibrium state of ZnIn_2_S_4_. The P1 model was therefore adopted to avoid artificial constraints and obtain fully optimized structural parameters.

In ab initio calculations, the system is generally considered at 0 K, representing the electronic ground state of the material. At this temperature, thermal vibrations, lattice expansion, and entropy contributions are neglected, allowing the intrinsic properties of the equilibrium crystal structure to be evaluated. Therefore, the calculated structural, electronic, optical, and mechanical properties correspond to the ideal zero‐temperature state of cubic ZnIn_2_S_4_. However, temperature‐dependent effects may cause variations in these properties under practical operating conditions.

The computational analysis was performed using a plane‐wave basis set with ultrasoft pseudopotentials to accurately describe the electronic states of ZnIn_2_S_4_. A cutoff energy of 500 eV was employed to ensure reliable convergence, while the Brillouin zone integration was carried out using the Monkhorst–Pack scheme with a 6 × 6 × 6*k*‐point grid, providing sufficient sampling accuracy for the cubic spinel structure [47]. The structural optimization was conducted using strict convergence criteria, including a maximum force tolerance of 0.03 eV Å^−1^, a stress tolerance of 0.02 GPa, a total energy convergence threshold of 2 × 10^−5 ^eV Å^−1^, maximum ionic displacement 10^−3 ^Å, and an Sele‐consistent field (SCF) energy convergence tolerance of 10^−6 ^eV Å^−1^ [48, 49, 50, 51]. High‐precision geometry optimization was performed to minimize numerical errors and obtain a reliable ground‐state structure. The optimized structure was subsequently used for the calculation of all investigated properties.

The structural characteristics were determined from the optimized lattice parameters, unit‐cell volume, and interatomic bond lengths. The mechanical stability and elastic response were examined through the calculation of independent elastic constants, followed by the determination of bulk modulus, shear modulus, Young’s modulus, Poisson’s ratio, Pugh’s ratio, elastic anisotropy, and hardness parameters using appropriate theoretical models, including the Voigt–Reuss–Hill (VRH) averaging approach [48, 49, 50, 51]. These mechanical parameters provide detailed information regarding the stiffness, ductility, brittleness, and directional dependence of the elastic behavior of ZnIn_2_S_4_. Furthermore, the dynamical stability of the optimized structure was evaluated through phonon dispersion calculations, where the absence of negative‐frequency phonon modes confirms the stability of the crystal against atomic displacement and lattice vibrations.

The electronic properties were analyzed through the electronic band structure, total density of states (DOS), and partial density of states (PDOS) calculations to understand the electronic states and orbital contributions near the Fermi level. The effective masses of electrons and holes were obtained from parabolic fitting of the energy dispersion near the conduction‐band minimum (CBM) and valence‐band maximum (VBM), respectively, providing insight into the carrier transport characteristics. Furthermore, the optoelectronic behavior was investigated through the calculation of the complex dielectric function, from which important optical parameters, including the refractive index, extinction coefficient, absorption coefficient, reflectivity, optical conductivity, and energy‐loss function, were derived.

This comprehensive first‐principles methodology integrates structural optimization, elastic analysis, carrier transport evaluation, and optical characterization to establish a detailed understanding of the fundamental properties of cubic spinel ZnIn_2_S_4_. The obtained results provide valuable insight into its potential suitability for semiconductor and optoelectronic applications.

## 3 Results and Discussion

### 3.1 Structural Optimization and Crystal Analysis

The geometry‐optimized crystal structures of cubic spinel ZnIn_2_S_4_ are presented in Figure 1. The corresponding fractional atomic coordinates of Zn, In, and S atoms before and after P1 relaxation are provided in Table 1, while the optimized structural parameters are summarized in Table 2. The conventional cubic spinel structure belonging to the $Fd\bar{3}m$ (No. 227) space group is presented in Figure 1a, whereas Figure 1b depicts its crystallographically equivalent primitive P1 representation employed in the present first‐principles calculations. Within the spinel lattice, S atoms constitute a cubic close‐packed framework, while Zn and In atoms occupy the tetrahedral and octahedral interstitial sites, respectively [13, 42]. The primitive P1 cell retains the local coordination environment and chemical bonding characteristics of the conventional cubic structure while removing the crystallographic symmetry constraints. Consequently, complete relaxation of both the lattice parameters and atomic positions was achieved during geometry optimization. The optimized structure preserves the characteristic spinel framework without any noticeable structural distortion, confirming that the primitive P1 model corresponds to a stable equilibrium configuration. This structural stability further validates the suitability of the primitive representation for accurately investigating the intrinsic structural, mechanical (elastic), electronic, effective mass, and optoelectronic properties of ZnIn_2_S_4_.

**FIGURE 1:**
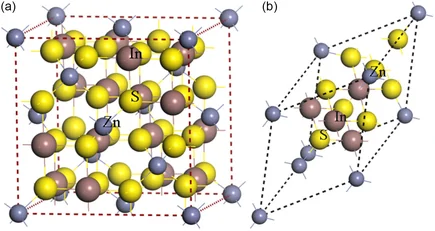
Optimized crystal structures of cubic spinel ZnIn_2_S_4_: (a) conventional cubic unit cell and (b) primitive P1 unit cell used for the DFT calculations.

**TABLE 1:** Fractional atomic coordinates of cubic spinel ZnIn_2_S_4_ before and after P1 relaxation.

Structure	Atoms	*x*	*y*	*z*
ZnIn_2_S_4_	Zn	0.25	0.25	0.25
	In	0.125	0.875	0.375
	S	0.37061	0.87061	0.37061
ZnIn_2_S_4_ (P1 Symmetry)	Zn	0.25	0.25	0.25
	In	1.125	−0.375	−0.375
	S	0.87152	−0.12848	0.87152

**TABLE 2:** Summary of structural, electronic, optical, mechanical characteristics, and career effective mass of ZnIn_2_S_4_.

Sample	*a,* Å	Δ*a,* *%*	***V*, Å** ^**3**^	***E***_**g**_, **eV**	Nature	*d* (Ca‐X), Å		References
ZnIn_2_S_4_ (spin‐polarized)	7.555^a^, (P1; equivalent conventional cell: 10.75 Å) 7.601^b^	0.605	304.991	1.289^a^	Indirec (L → G)	S─Zn S─In	2.377^a^ 2.634^a^	^a^ Present work ^b^Ref [46]
ZnIn_2_S_4_ (nonspin‐polarized)	7.631^a^, (P1; equivalent conventional cell: 10.75 Å)	0.401	314.211	1.123^a^	Indirect (L → G)	S─Zn S─In	2.415^a^ 2.653^a^	

The optimized primitive lattice parameterof cubic ZnIn_2_S_4_ was calculated to be 7.555 Å, which is in close agreement with the reported value of 7.601 Å, corresponding to a deviation of only 0.605% [13, 42]. The optimized primitive unit‐cell volume was obtained as 304.991 Å^3^, indicating that the structure reaches a stable equilibrium configuration after geometry optimization. The close agreement between the calculated and reported structural parameters confirms the reliability of the spin‐polarized GGA‐PBE methodology used in the present study [42]. The small deviation may be attributed to the approximate treatment of exchange–correlation interactions within the GGA functional, which is commonly observed in first‐principles calculations. The calculated Zn─S and In─S bond lengths were found to be 2.377 and 2.634 Å, respectively, confirming the stability of the optimized spinel framework.

To examine the effect of spin polarization on the structural and electronic characteristics, additional calculations were performed using the nonspin‐polarized GGA‐PBE approach. The nonspin‐polarized calculation resulted in a primitive lattice parameter of 7.631 Å and a unit‐cell volume of 314.211 Å^3^, showing close agreement with the reported structural parameters. The corresponding Zn─S and In─S bond lengths were calculated as 2.415 and 2.653 Å, respectively. Furthermore, the nonspin‐polarized calculation yielded an indirect bandgap of 1.123 eV, with the VBM and CBM located at different *k*‐points along the L → G direction. The small differences between spin‐polarized and nonspin‐polarized results indicate that spin polarization has a negligible influence on the structural and electronic properties of cubic ZnIn_2_S_4_. Therefore, the nonspin‐polarized GGA‐PBE approach was adopted for the subsequent analysis of the electronic, optical, mechanical, and transport properties.

The relatively shorter Zn─S bond indicates stronger bonding interactions between Zn and S atoms within the tetrahedral coordination environment, whereas the comparatively longer In─S bond arises from the larger ionic radius of In together with its octahedral coordination in the spinel framework. The optimized structural parameters, including the primitive lattice parameter, unit‐cell volume, and metal–sulfur bond lengths, demonstrate the structural integrity of the primitive P1 model and confirm the excellent stability of the optimized crystal structure. Moreover, the close agreement with previously reported structural data provides confidence in the accuracy of the computational approach adopted in this work and establishes a dependable basis for the subsequent analysis of the mechanical (elastic), electronic, effective mass, and optoelectronic properties of cubic spinel ZnIn_2_S_4_.

### 3.2 Electronic Band Structure and DOS

The electronic band structure provides fundamental insight into the electrical conductivity, optical response, and carrier transport behavior of semiconductor materials. Within the framework of DFT, the electronic properties are described by the Kohn–Sham equations, where electrons move in an effective potential given by [52]:

$$V_{S}\left(r\right)=V\left(r\right)+\int \frac{e^{2}n(r\prime )}{\left|r-r\prime \right|}\;d^{3}r\prime +V_{\mathrm{XC}}\left[n\left(r\right)\right]$$

where *V*(*r*) denotes the external ionic potential, the second term represents the Hartree potential accounting for electron–electron Coulomb interactions, and *V*_XC_ is the exchange–correlation potential. The self‐consistent solution of the Kohn–Sham equations yields the electronic energy eigenvalues, from which the band structure and DOS are obtained.

The electronic band structures of primitive cubic spinel ZnIn_2_S_4_ were calculated along the W–L–G–X–W–K high‐symmetry path of the Brillouin zone using spin‐polarized GGA‐PBE and nonspin‐polarized GGA‐PBE approaches, as presented in Figure 2a,b, respectively. The band structures provide information about the energy dispersion of electrons with respect to crystal momentum and are essential for understanding the electronic transport behavior and bandgap characteristics of the material. Both approaches predict an indirect semiconducting nature for cubic ZnIn_2_S_4_, where the VBM and CBM occur at different *k*‐points. The calculated indirect bandgaps are 1.289 eV for spin‐polarized GGA‐PBE and 1.123 eV for nonspin‐polarized GGA‐PBE, respectively. The difference of 0.166 eV between the two calculations indicates that spin polarization has a minor influence on the electronic bandgap of ZnIn_2_S_4_, confirming its weak magnetic contribution.

**FIGURE 2:**
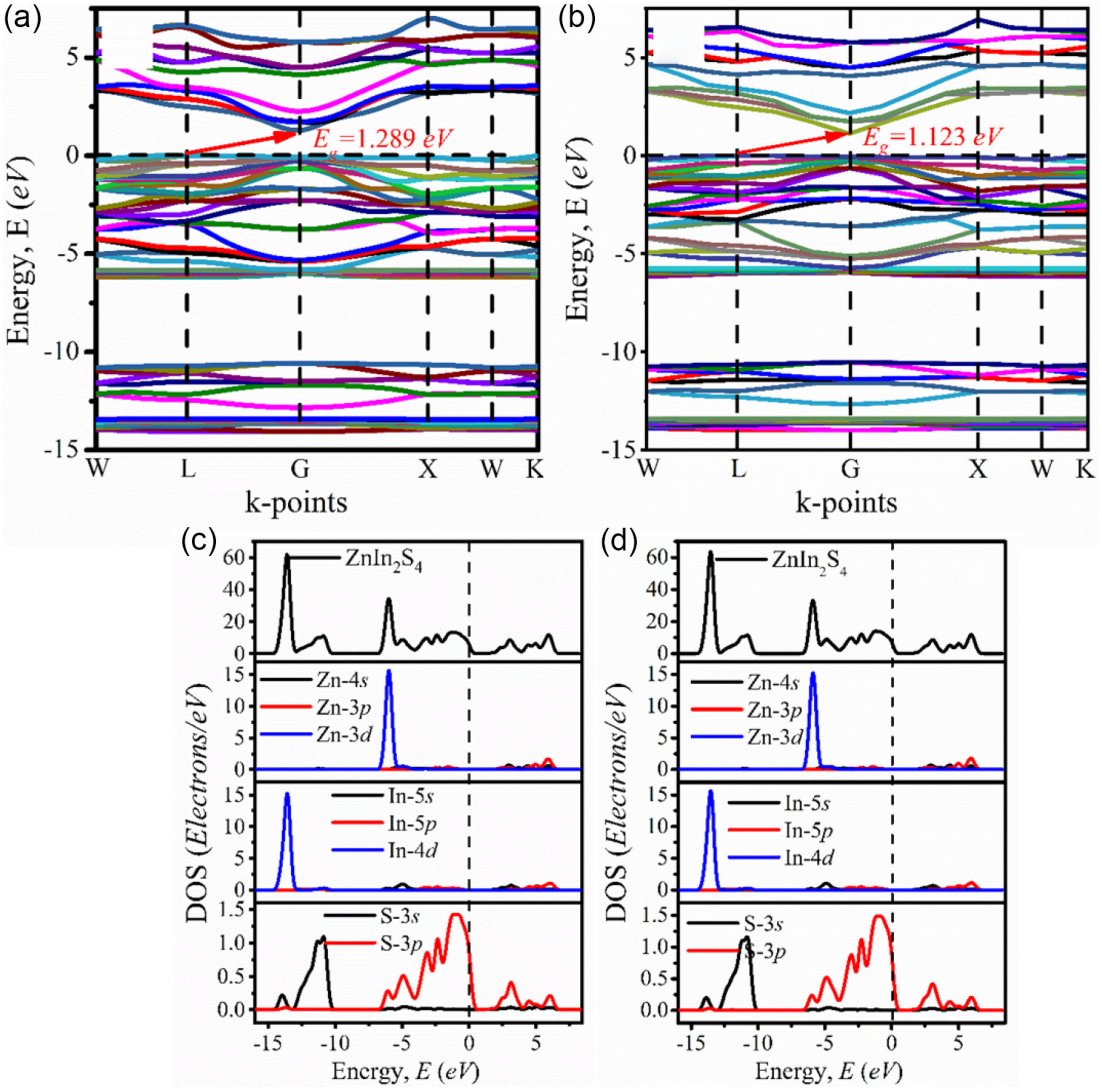
Electronic properties of cubic spinel ZnIn_2_S_4_: calculated electronic band structures along the W–L–G–X–W–K high‐symmetry path using (a) spin‐polarized GGA‐PBE with an indirect bandgap of 1.289 eV and (b) nonspin‐polarized GGA‐PBE with an indirect bandgap of 1.123 eV; total and orbital‐resolved partial density of states (DOS/PDOS) calculated using (c) spin‐polarized GGA‐PBE and (d) nonspin‐polarized GGA‐PBE. The dashed horizontal line represents the Fermi level.

The calculated bandgap values are obtained using the PBE exchange–correlation functional, which is known to underestimate semiconductor bandgaps due to the self‐interaction error and the discontinuity problem in the exchange–correlation potential. Therefore, the predicted PBE bandgaps should be considered as lower limit estimates of the actual electronic bandgap. This limitation can influence the quantitative prediction of optical properties, particularly the absorption edge, dielectric response, and optical conductivity, because these properties are directly related to electronic transitions across the bandgap. A smaller PBE bandgap may result in a slight redshift of the calculated optical absorption onset compared with experimentally measured or higher level theoretical values. However, the qualitative interpretation of the optical response, including the active transition regions and overall spectral behavior, remains reliable because the orbital characteristics and electronic transition mechanisms are preserved.

The electronic states near the band edges originate from the hybridization of Zn, In, and S atomic orbitals within the spinel framework. The upper valence‐band region is mainly contributed by Zn 3*d* and S 3*p* states through strong *p*–*d* hybridization, whereas the CBM is predominantly derived from In 5*s*, In 5*p*, and Zn 4*s* states through *s*–*p* orbital interactions. These orbital contributions determine the position of the VBM and CBM and govern the carrier transport and optical excitation behavior of ZnIn_2_S_4_. Therefore, although the PBE functional introduces a quantitative underestimation of the bandgap, the calculated electronic structure provides valuable insight into the intrinsic semiconductor characteristics and supports the evaluation of ZnIn_2_S_4_ for optoelectronic applications.

The dashed horizontal line at 0 eV represents the Fermi level, which lies within the forbidden energy gap, confirming the semiconducting character of ZnIn_2_S_4_. All states below the Fermi level are occupied, while those above it remain empty under equilibrium conditions. Upon optical excitation, electrons are promoted from the hybridized valence‐band states to the conduction‐band states, generating electron–hole pairs that contribute to electrical conduction and optical transitions. Although the GGA‐calculated bandgap is smaller than the experimental value, owing to the well‐known underestimation of semilocal DFT, the calculated band structure reliably captures the electronic dispersion and orbital interactions of ZnIn_2_S_4_, providing a solid basis for the subsequent DOS and optical property analyses.

To further elucidate the electronic structure of cubic ZnIn_2_S_4_ using spin‐polarized GGA‐PBE and nonspin‐polarized GGA‐PBE approaches, the total DOS and orbital‐resolved PDOS were calculated and are presented in Figure 2c,d, respectively. Both calculations were performed using the same optimized crystal structure, allowing a direct comparison of the influence of spin polarization on the electronic states. The DOS profiles obtained from both approaches show similar distributions of electronic states, indicating that spin polarization has a negligible effect on the overall electronic structure of ZnIn_2_S_4_. While the electronic band structure describes the dispersion of energy bands, the DOS provides the distribution of available electronic states as a function of energy, and the PDOS identifies the contributions of individual atomic orbitals. Consequently, DOS and PDOS analyses are indispensable for understanding the origin of the valence and conduction bands, the nature of chemical bonding, orbital hybridization, charge‐transfer mechanisms, and the electronic transitions responsible for the optical and transport properties of semiconductors.

The calculated DOS exhibits a distinct forbidden energy region around the Fermi level (0 eV), confirming the semiconducting character of ZnIn_2_S_4_. The absence of electronic states at the Fermi level indicates that the valence band is completely occupied, whereas the conduction band remains empty under equilibrium conditions. Therefore, electrons can contribute to electrical conduction only after absorbing sufficient thermal or optical energy to overcome the bandgap.

The lower valence band, extending from approximately −15 to −7 eV, is mainly composed of localized In 4*d* and S 3*s* orbitals. The intense peaks near −13.53 eV arise predominantly from the In 4*d* states, whereas those around −13.6 and −10.8 eV originate mainly from the S 3*s* orbitals. These orbitals possess high binding energies and remain strongly localized around their respective atoms, representing deep core (or semicore) electronic states. Their limited overlap indicates only weak hybridization, and consequently they contribute little to electrical conduction or optical excitation but play an important role in maintaining the structural stability of the crystal.

The upper valence band (approximately −7 to 0 eV) is dominated by S 3*p* states with significant contributions from Zn 3*d* orbitals, particularly around −6.3 and −5.5 eV. The substantial overlap between these orbitals demonstrates strong Zn 3*d*–S 3*p* *p*–*d* hybridization, reflecting pronounced covalent interaction between Zn and S atoms. This hybridization broadens the valence band, strengthens the Zn─S chemical bonds, and determines the position of the VBM. Since the electronic states immediately below the Fermi level are primarily derived from S 3*p* orbitals, the photogenerated holes predominantly possess sulfur *p*‐orbital character.

Above the Fermi level, the conduction band is mainly formed by In 5*s*, In 5*p*, and Zn 4*s* orbitals, with smaller contributions from Zn 3*p* and S 3*p* states. The overlap among these orbitals reveals pronounced *s*–*p* hybridization, producing delocalized antibonding states that constitute the conduction‐band edge. Compared with the localized core states, these conduction‐band states are spatially extended throughout the crystal lattice, facilitating electron transport after photoexcitation and enhancing carrier mobility.

Overall, the electronic structure of ZnIn_2_S_4_ is governed by two principal hybridization mechanisms: Zn 3*d*–S 3*p* *p*–*d* hybridization, which controls the valence‐band characteristics, and In 5*s*/In 5*p*–Zn 4*s* *s*–*p* hybridization, which determines the conduction‐band properties. The deep In 4*d* and S 3*s* orbitals remain localized far below the Fermi level and have negligible influence on the electronic transitions near the bandgap. These hybridized electronic states collectively govern the electronic, optical, and transport behavior of ZnIn_2_S_4_.

The DOS and PDOS results further indicate that ZnIn_2_S_4_ possesses a favorable electronic structure for photoinduced energy conversion. The moderate bandgap together with the strong orbital hybridization enables efficient excitation of electrons from the Zn 3*d*/S 3*p*‐derived valence band to the In 5*s*/In 5*p*‐derived conduction band under visible‐light irradiation. The delocalized conduction‐band states facilitate rapid migration of excited electrons, while the indirect bandgap nature suppresses rapid electron–hole recombination, thereby increasing the lifetime of photogenerated charge carriers. Such efficient carrier generation, separation, and transport are highly desirable for photocatalytic reactions, where electrons and holes migrate to the material surface to participate in reduction and oxidation processes, respectively.

Furthermore, the calculated electronic structure indicates that ZnIn_2_S_4_ possesses favorable semiconductor characteristics for solar‐responsive and optoelectronic applications. The moderate bandgap enables effective interaction with visible‐light radiation, while the suitable electronic structure and carrier transport properties support efficient photo‐induced charge generation and transport processes. These characteristics highlight the potential of ZnIn_2_S_4_ for applications such as photodetectors, photovoltaic‐related systems, and other light‐responsive semiconductor technologies. However, practical device performance depends on additional factors, including band‐edge alignment, defect states, carrier lifetime, interface effects, and experimental optimization.

In addition, the favorable band dispersion, efficient orbital hybridization, and semiconducting bandgap make ZnIn_2_S_4_ attractive for a broad range of electronic and optoelectronic applications, including photodetectors, photovoltaic absorbers, phototransistors, photoconductive devices, visible‐light sensors, solar‐energy harvesting systems, and other semiconductor devices, where efficient light absorption, charge separation, and carrier transport are essential for high‐performance operation [53].

### 3.3 Carrier Effective Mass and Carrier Mobility Analysis

The carrier effective mass is a fundamental parameter for evaluating the transport behavior of semiconductors because it determines how easily electrons and holes move through a crystal under an applied electric field. It provides essential information about carrier mobility and transport behavior, as a smaller effective mass generally indicates easier carrier movement and higher transport efficiency. Therefore, effective mass analysis is crucial for evaluating the electronic transport characteristics of ZnIn_2_S_4_. In a crystalline solid, charge carriers do not behave as free particles due to the periodic potential created by the atomic lattice. Instead, they move as quasiparticles with an effective mass (m*), which depends on the curvature of the electronic bands near the CBM and VBM. The effective mass is calculated by the Bloch relation [54, 55]:

$$m*=\hbar^{2}{\left(\frac{\partial^{2}E}{\partial k^{2}}\right)}^{-1}$$

where m* is the effective mass, ℏ is the reduced Planck constant, *E* is the electronic energy, and *k* is the crystal wave vector. A highly dispersive band possesses a larger curvature (*d* ^2^ *E*/*dk* ^2^), resulting in a smaller effective mass, whereas a flatter band corresponds to a larger effective mass. Therefore, materials with smaller carrier effective masses generally exhibit superior electrical transport properties because their charge carriers can be accelerated more readily.

In this work, carrier mobility was evaluated within the constant relaxation time approximation, which assumes a uniform carrier scattering time for comparative analysis. Although the absolute mobility depends on the relaxation time, this approach provides valuable insight into the intrinsic transport capability determined by electronic band dispersion and carrier effective masses. The calculated mobility should therefore be interpreted as a relative indicator of carrier transport performance rather than a direct prediction of experimentally measured mobility.

The parabolic fitting of the band edges for hole and electron effective mass determination is presented in Figure 3, and the calculated carrier effective masses of cubic spinel ZnIn_2_S_4_ are summarized in Table 3. The electron effective mass is 0.129*m*_e_, while the hole effective mass is −1.970*m*_e_. The negative sign of the hole effective mass arises from the negative curvature of the valence band near the VBM and is a characteristic feature of hole transport in semiconductors [57]. Physically, holes are treated as positively charged carriers with a positive effective mass equal to the absolute value of the calculated quantity. Comparing the magnitudes, the electron effective mass is nearly 15 times smaller than that of the hole, indicating that the conduction band is considerably more dispersive than the valence band.

**FIGURE 3:**
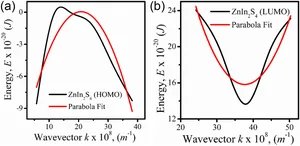
Parabolic fitting of the band edges used to determine the (a) hole and (b) electron effective masses of cubic spinel ZnIn_2_S_4_.

**TABLE 3:** Polycrystalline elastic properties, elastic anisotropy parameters, eigenvalues of the stiffness matrix, and estimated carrier mobility of cubic ZnIn_2_S_4_ calculated using the GGA‐PBE functional.

Category	Parameter	Symbol	Unit	Value	Reference
Polycrystalline elastic moduli (Voigt)	Bulk modulus	*K*_V_	GPa	75.896^a^ 66.04^b^ 18.58^c^ (Hexagonal)	^a^Present ^b^ Ref [56] ^c^ Ref [53]
	Young’s modulus	*E*_V_	GPa	99.376^a^ 78.75^b^ 21.73^c^ (Hexagonal)	
	Shear modulus	*G*_V_	GPa	38.765^a^ 31.97^b^ 8.33^c^ (Hexagonal)	
	Poisson’s ratio	*ν*_V_	—	0.28177	
Polycrystalline elastic moduli (Reuss)	Bulk modulus	*K*_R_	GPa	75.896	
	Young’s modulus	*E*_R_	GPa	97.692	
	Shear modulus	*G*_R_	GPa	37.998	
	Poisson’s ratio	*ν*_R_	—	0.28547	
Polycrystalline elastic moduli (Hill)	Bulk modulus	*K*_H_	GPa	75.896	
	Young’s modulus	*E*_H_	GPa	98.535	
	Shear modulus	*G*_H_	GPa	38.382	
	Poisson’s ratio	*ν*_H_	—	0.28362	
Elastic anisotropy	Minimum Young’s modulus	*E*_min_	GPa	84.81	
	Maximum Young’s modulus	*E*_max_	GPa	108.70	
	Young’s modulus anisotropy	*A*_E_	—	1.282	
	Minimum linear compressibility	*β*_min_	TPa ^−1^	4.392	
	Maximum linear compressibility	*β*_max_	TPa ^−1^	4.392	
	Linear compressibility anisotropy	*A* *_β_*	—	1.0000	
	Minimum shear modulus	*G*_min_	GPa	32.278	
	Maximum shear modulus	*G*_max_	GPa	43.090	
	Shear modulus anisotropy	*A*_G_	—	1.335	
	Minimum Poisson’s ratio	*ν*_min_	—	0.17832	
	Maximum Poisson’s ratio	*ν*_max_	—	0.37568	
	Poisson’s ratio anisotropy	*A*_ν_	—	2.1068	
Eigenvalues of the stiffness matrix	First eigenvalue	*λ*_1_	GPa	43.090	
	Second eigenvalue	*λ*_2_	GPa	43.090	
	Third eigenvalue	*λ*_3_	GPa	43.090	
	Fourth eigenvalue	*λ*_4_	GPa	64.555	
	Fifth eigenvalue	*λ*_5_	GPa	64.555	
	Sixth eigenvalue	*λ*_6_	GPa	227.690	
Carrier transport	Electron effective mass	$m_{e}^{*}/m_{e}$	—	0.129	
	Hole effective mass	$m_{h}^{*}/m_{e}$	—	−1.970	
	Electron mobility	*μ*_e_	cm^2 ^V ^−1 ^s ^−1^	136.3	
	Hole mobility	*μ*_h_	cm^2 ^V ^−1 ^s ^−1^	8.91	

The significantly smaller electron effective mass suggests that electrons experience less inertia while moving through the crystal lattice and can therefore respond more efficiently to external electric fields. In contrast, the relatively large hole effective mass indicates that the valence‐band states are more localized, resulting in slower hole transport [58]. This asymmetry in carrier effective masses implies that electrical conduction in ZnIn_2_S_4_ is expected to be dominated by electrons rather than holes. The low electron effective mass also reflects the favorable dispersion of the conduction band, which is advantageous for efficient carrier transport and reduced scattering during electronic conduction. Consequently, the calculated effective masses indicate that ZnIn_2_S_4_ possesses excellent electron transport characteristics, providing a strong basis for the subsequent carrier mobility analysis.

Carrier mobility is one of the most important transport parameters of semiconductors because it describes the ability of charge carriers to move through a crystal under the influence of an external electric field. It determines the drift velocity of electrons and holes and directly affects the electrical conductivity, carrier diffusion length, switching speed, and photoelectric performance of semiconductor devices. In general, high carrier mobility enables rapid charge transport, minimizes resistive losses, and improves the efficiency of charge collection in electronic and optoelectronic systems [59, 60, 61].

Within the relaxation‐time approximation, the carrier mobility is related to the carrier effective mass through [62]

$$\mu =\frac{e\tau }{m*}$$

where *μ* is the carrier mobility, *e* is the elementary charge, *τ* is the carrier relaxation time, and m* is the carrier effective mass. The relaxation time represents the average time interval between two successive scattering events experienced by a charge carrier during its motion through the crystal lattice. Carrier scattering may arise from lattice vibrations (phonons), crystal imperfections, impurities, grain boundaries, or defects, all of which reduce carrier mobility. In the present study, a constant relaxation time of *τ* = 1 × 10^−14 ^s was adopted to estimate the carrier mobility [60, 62]. According to the above equation, carrier mobility is directly proportional to the relaxation time and inversely proportional to the effective mass. Therefore, carriers possessing a smaller effective mass and longer relaxation time generally exhibit superior transport characteristics.

The calculated carrier mobilities of ZnIn_2_S_4_ are summarized in Table 3. The electron mobility is 136.3 cm^2 ^V^−1 ^s^−1^, whereas the hole mobility is 8.91 cm^2 ^V^−1 ^s^−1^. These values are consistent with the effective mass analysis discussed previously, where the electron effective mass (0.129*m*_e_) is considerably smaller than the magnitude of the hole effective mass (1.970*m*_e_). Because mobility is inversely proportional to the effective mass, electrons require less energy to accelerate within the periodic crystal potential and can therefore move much more rapidly than holes. Conversely, the larger hole effective mass results in significantly lower hole mobility, indicating slower hole transport through the crystal lattice.

The electron mobility is approximately 15 times higher than the hole mobility, indicating that electrical conduction in ZnIn_2_S_4_ is predominantly governed by electrons. This behavior is also consistent with the electronic band structure, where the conduction band exhibits greater dispersion than the valence band, leading to a smaller electron effective mass and enhanced carrier transport. The high electron mobility suggests that photoexcited electrons can migrate efficiently through the crystal with reduced scattering losses, while the comparatively lower hole mobility results in slower hole transport. Such asymmetric carrier transport promotes spatial separation of electrons and holes, thereby reducing their probability of recombination and extending the lifetime of photogenerated charge carriers.

The combination of a low electron effective mass and high electron mobility demonstrates that ZnIn_2_S_4_ possesses excellent electron transport capability. Rapid electron migration facilitates efficient charge transfer within the crystal and improves carrier collection efficiency under electrical or optical excitation. These favorable transport characteristics are expected to enhance the electrical conductivity, photoresponse, and overall transport performance of the material, making ZnIn_2_S_4_ an attractive semiconductor for high‐performance electronic devices [63].

The excellent carrier transport properties indicate that ZnIn_2_S_4_ possesses favorable intrinsic characteristics for photoresponsive and optoelectronic applications. The high carrier mobility can facilitate efficient charge transport, while the moderate bandgap supports effective interaction with visible‐light radiation. These electronic and transport features, combined with the calculated optical response, suggest potential relevance for semiconductor‐based photodetectors, photovoltaic‐related systems, and other optoelectronic devices. However, practical device performance depends on additional factors, including defect states, carrier lifetime, interface effects, and experimental device optimization.

### 3.4 Optical Behavior and Spectral Response

The optical properties of a semiconductor are intrinsically governed by its electronic structure and provide valuable insight into the interaction between electromagnetic radiation and matter. These properties arise from electronic transitions between the occupied valence‐band states and the unoccupied conduction‐band states upon photon irradiation and are therefore closely related to the band structure and DOS. The frequency‐dependent optical response is characterized by the complex dielectric function, from which other important optical constants, including the refractive index, extinction coefficient, absorption coefficient, reflectivity, photoconductivity, and energy loss function, are derived. Collectively, these optical parameters describe the polarization, light absorption, propagation, reflection, charge‐carrying capability, and energy dissipation of the material over a wide photon‐energy range. In the present study, the optical behavior of cubic spinel ZnIn_2_S_4_ was investigated within the GGA‐PBE framework, and the calculated spectra reveal a pronounced response extending from the visible to the ultraviolet region [64, 65].

#### 3.4.1 Real and Imaginary Dielectric Constant

The dielectric function is one of the most fundamental optical parameters for describing the interaction between electromagnetic radiation and a material. It is represented by the complex dielectric function [66, 67]

$$\varepsilon (\omega )=\varepsilon_{r}(\omega )+i\varepsilon_{i}(\omega )$$

where *ε*_r_(*ω*) and *ε*_*i*_(*ω*) denote the real and imaginary parts of the dielectric function, respectively. The real dielectric constant, *ε*_r_(*ω*), describes the polarization and energy‐storage capability of the material under an external electromagnetic field, whereas the imaginary dielectric constant, *ε*_*i*_(*ω*), represents the absorption of incident photons due to interband electronic transitions from the valence band to the conduction band. Therefore, these two quantities provide comprehensive information about the optical response and electronic excitation behavior of semiconductors.

The calculated real and imaginary dielectric constants of cubic ZnIn_2_S_4_ are presented in Figure 4a. At zero photon energy, the static dielectric constant, *ε*_r_(0), is 7.66, indicating relatively strong electronic polarization under a static electric field. As the photon energy increases, *ε*_r_(*ω*) gradually rises and reaches its maximum value of 10.21 at approximately 3.18 eV, which lies within the visible‐light region. This pronounced peak originates from strong interband electronic transitions between the hybridized Zn 3*d*/S 3*p* states forming the upper valence band and the In 5*s*/In 5*p* states constituting the conduction band. Beyond this energy, the real dielectric constant decreases rapidly because the polarization capability of the electron cloud diminishes as higher energy photons excite electrons more efficiently into the conduction band.

**FIGURE 4:**
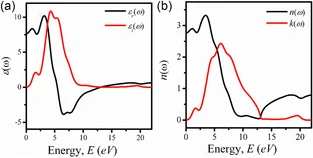
Optical dielectric response of cubic spinel ZnIn_2_S_4_: (a) real and imaginary parts of the dielectric function and (b) refractive index and extinction coefficient.

With further increasing photon energy, *ε*_r_(*ω*) becomes negative in the energy range of approximately 5.5–8.5 eV. A negative dielectric constant indicates that the incident electromagnetic wave cannot propagate efficiently through the material because the collective oscillation of electrons dominates the optical response, leading to a metallic‐like reflection behavior within this energy range. This behavior is associated with the plasma resonance of the electron gas and is consistent with the first and second plasma energies observed near 5.45 and 12.90 eV, respectively. At higher photon energies, the dielectric constant gradually approaches a nearly constant positive value, indicating reduced polarization as the contribution of interband transitions decreases.

The imaginary dielectric constant, *ε*_*i*_(*ω*), directly reflects the optical absorption resulting from electronic transitions. As shown in Figure 4a, the static value of *ε*_*i*_(*ω*) is 0.0149, confirming negligible absorption in the absence of photon excitation. The spectrum begins to increase sharply above the bandgap energy, indicating the onset of interband transitions from the occupied valence‐band states to the empty conduction‐band states. The maximum value of 11.0 occurs at approximately 4.3 eV, corresponding to the highest probability of optical transitions. This strong absorption peak mainly originates from transitions between the hybridized Zn 3*d*/S 3*p* valence‐band states and the In 5*s*/In 5*p* conduction‐band states identified in the electronic structure analysis. Beyond the maximum, *ε*_*i*_(*ω*) decreases gradually because the transition probability becomes lower at higher photon energies as the available electronic states are progressively depleted.

The imaginary dielectric constant, *ε*_*i*_(*ω*), represents the absorptive component of the dielectric function and describes the absorption of electromagnetic radiation through interband electronic transitions. It is directly associated with the excitation of electrons from occupied valence‐band states to unoccupied conduction‐band states and therefore determines the optical absorption behavior of a material. The imaginary part of the dielectric function is calculated using Fermi’s Golden Rule as [66, 68]

$$\varepsilon_{i}\left(\omega \right)=\frac{4\pi^{2}e^{2}}{m^{2}\omega^{2}}\sum_{c\text{,}v}\int_{\mathrm{BZ}}\frac{d^{3}k}{{\left(2\pi \right)}^{3}}{\left|\left\langle c,k|\hat{v}|v,k\right\rangle \right|}^{2}\delta \left(E_{c}(k)-E_{v}(k)-\hbar \omega \right)$$

where *e* is the elementary charge, *m* is the electron mass, *ω* is the angular frequency of the incident photon, *c* and *v* represent the conduction‐ and valence‐band states, respectively, *E* *_c_* (*k*) and *E* *_v_* (*k*) are their corresponding energies, $\hat{v}$ is the velocity operator, and the delta function ensures conservation of energy during the optical transition. Consequently, *ε*_*i*_(*ω*) is proportional to the probability of photon‐induced electronic transitions between occupied and unoccupied states.

The calculated imaginary dielectric constant of cubic ZnIn_2_S_4_ is shown in Figure 4a. At zero photon energy, *ε*_*i*_(0) is 0.0149, indicating negligible optical absorption in the absence of incident photons. As the photon energy increases beyond the bandgap energy, *ε*_*i*_(*ω*) rises sharply, marking the onset of interband electronic transitions from the hybridized Zn 3*d*/S 3*p* valence‐band states to the In 5*s*/In 5*p* conduction‐band states. The imaginary dielectric constant reaches its maximum value of 11.0 at approximately 4.3 eV, corresponding to the highest transition probability between the occupied and unoccupied electronic states. This pronounced peak originates from the high density of available states and strong optical transition matrix elements arising from the orbital hybridization near the band edges.

Beyond 4.3 eV, *ε*_*i*_(*ω*) decreases gradually with increasing photon energy, indicating a reduction in the probability of interband transitions as the density of available electronic states and transition probabilities diminish. The broad absorption profile extending from the visible to the ultraviolet region demonstrates the ability of ZnIn_2_S_4_ to efficiently absorb photons over a wide spectral range. Combined with the relatively large magnitude of *ε*_*i*_(*ω*), this behavior suggests efficient photoexcitation and generation of electron–hole pairs, making ZnIn_2_S_4_ a promising material for light‐harvesting and photoresponsive semiconductor applications.

Overall, the relatively high static dielectric constant, the strong dielectric response in the visible region, and the pronounced interband transition peaks demonstrate that ZnIn_2_S_4_ possesses excellent optical polarization and photon‐absorption characteristics.

#### 3.4.2 Refractive Index and Extinction Coefficient

The refractive index is one of the fundamental optical constants that describes the propagation of electromagnetic waves through a material. It is defined as the ratio of the speed of light in vacuum to that in the material and determines the degree to which light is slowed and refracted upon entering the medium. The refractive index governs important optical phenomena such as light transmission, refraction, optical confinement, and waveguiding, making it a key parameter for designing optical and optoelectronic devices. The refractive index is directly related to the complex dielectric function and is expressed as [66]

$$n\left(\omega \right)={\left[\frac{\sqrt{\varepsilon_{r}^{2}(\omega )+\varepsilon_{i}^{2}(\omega )}+\varepsilon_{r}\left(\omega \right)}{2}\right]}^{1/2}$$

where *ε*_r_(*ω*) and *ε*_*i*_(*ω*) are the real and imaginary parts of the dielectric function, respectively. A larger refractive index generally indicates stronger interaction between incident light and the material due to greater electronic polarization.

The calculated refractive index spectrum of cubic ZnIn_2_S_4_ is shown in Figure 4b. At zero photon energy, the static refractive index is 2.76, indicating a relatively high optical density and strong polarization of the electronic cloud under low‐energy electromagnetic radiation. As the photon energy increases, the refractive index gradually rises and reaches a maximum value of 3.34 at approximately 3.44 eV. This pronounced peak lies within the visible‐light region and is attributed to strong interband electronic transitions from the hybridized Zn 3*d*/S 3*p* valence‐band states to the In 5*s*/In 5*p* conduction‐band states. The high density of available electronic states and enhanced transition probability near this energy lead to increased electronic polarization, resulting in the maximum refractive index.

Beyond 3.44 eV, the refractive index decreases rapidly with increasing photon energy. This behavior is associated with the reduction in electronic polarization as incident photons possess sufficient energy to excite electrons efficiently into higher energy conduction‐band states. Consequently, the ability of the electron cloud to respond to the oscillating electromagnetic field decreases, producing the observed decline in the refractive index. At higher photon energies, the refractive index becomes nearly constant with only slight variations, indicating that the optical response is dominated by high‐energy electronic excitations.

The relatively high static refractive index together with the strong optical response in the visible region demonstrates that ZnIn_2_S_4_ exhibits excellent light–matter interaction and optical confinement characteristics.

The extinction coefficient, *k*(*ω*), describes the attenuation of electromagnetic waves as they propagate through a material due to optical absorption. Physically, it represents the imaginary component of the complex refractive index and quantifies the loss of light intensity caused by electronic excitations within the crystal. A larger extinction coefficient corresponds to stronger absorption of incident photons and reduced optical transmission. Since the extinction coefficient is directly associated with photon absorption, it plays a vital role in evaluating semiconductor materials for light‐harvesting, photovoltaic, and photocatalytic applications. The extinction coefficient is related to the complex dielectric function by [69, 70]

$$k\left(\omega \right)={\left[\frac{\sqrt{\varepsilon_{r}^{2}(\omega )+\varepsilon_{i}^{2}(\omega )}-\varepsilon_{r}\left(\omega \right)}{2}\right]}^{1/2}$$

where *ε*_r_(*ω*) and *ε*_*i*_(*ω*) are the real and imaginary parts of the dielectric function, respectively. The extinction coefficient increases when the probability of interband electronic transitions becomes significant, leading to enhanced optical absorption.

The calculated extinction coefficient spectrum of cubic ZnIn_2_S_4_ is presented in Figure 4b. At low photon energies, *k*(*ω*) remains close to zero, indicating negligible optical attenuation because the incident photon energy is insufficient to induce significant interband electronic transitions. As the photon energy exceeds the bandgap energy, the extinction coefficient increases rapidly, reflecting the onset of strong photon absorption resulting from electronic excitations between the occupied valence‐band states and the unoccupied conduction‐band states.

The extinction coefficient reaches its maximum value of 2.45 at approximately 6.1 eV, where the probability of interband transitions is highest. This pronounced peak is mainly attributed to transitions from the hybridized Zn 3*d*/S 3*p* states in the upper valence band to the In 5*s*/In 5*p* states in the conduction band, consistent with the electronic band structure and DOS analyses. The strong orbital hybridization near the band edges enhances the optical transition probability, giving rise to the observed maximum in the extinction spectrum.

Beyond 6.1 eV, the extinction coefficient decreases gradually with increasing photon energy, indicating a reduction in optical attenuation as the transition probability between occupied and unoccupied states decreases. At higher photon energies, only weak features are observed, suggesting that most of the dominant interband transitions have already occurred, and the optical absorption gradually diminishes.

#### 3.4.3 Absorption Coefficient and Reflectivity

The absorption coefficient, *α*(*ω*), is one of the most important optical parameters for evaluating the light‐harvesting capability of semiconductors. It measures the rate at which the intensity of incident light decreases as it propagates through a material due to photon absorption. A high absorption coefficient indicates that the material can efficiently absorb incident photons within a short penetration depth, resulting in the generation of a large number of electron–hole pairs. Consequently, the absorption coefficient is a key parameter for assessing the suitability of materials for photovoltaic, photocatalytic, and optoelectronic applications. It is expressed as [66, 71, 72]

$$\alpha \left(\omega \right)=\frac{2\omega k(\omega )}{c}$$

where *ω* is the angular frequency of the incident photon, *k*(*ω*) is the extinction coefficient, and *c* is the speed of light in vacuum.

The calculated absorption spectrum of cubic ZnIn_2_S_4_ is shown in Figure 5a. As expected, the absorption coefficient is nearly zero below the bandgap energy because photons with energies lower than the bandgap cannot excite electrons from the valence band to the conduction band. Once the photon energy exceeds the bandgap, the absorption coefficient increases rapidly, marking the onset of strong interband electronic transitions. This behavior is consistent with the electronic band structure and DOS analyses, where electrons are excited from the hybridized Zn 3*d*/S 3*p* valence‐band states to the In 5*s*/In 5*p* conduction‐band states.

**FIGURE 5:**
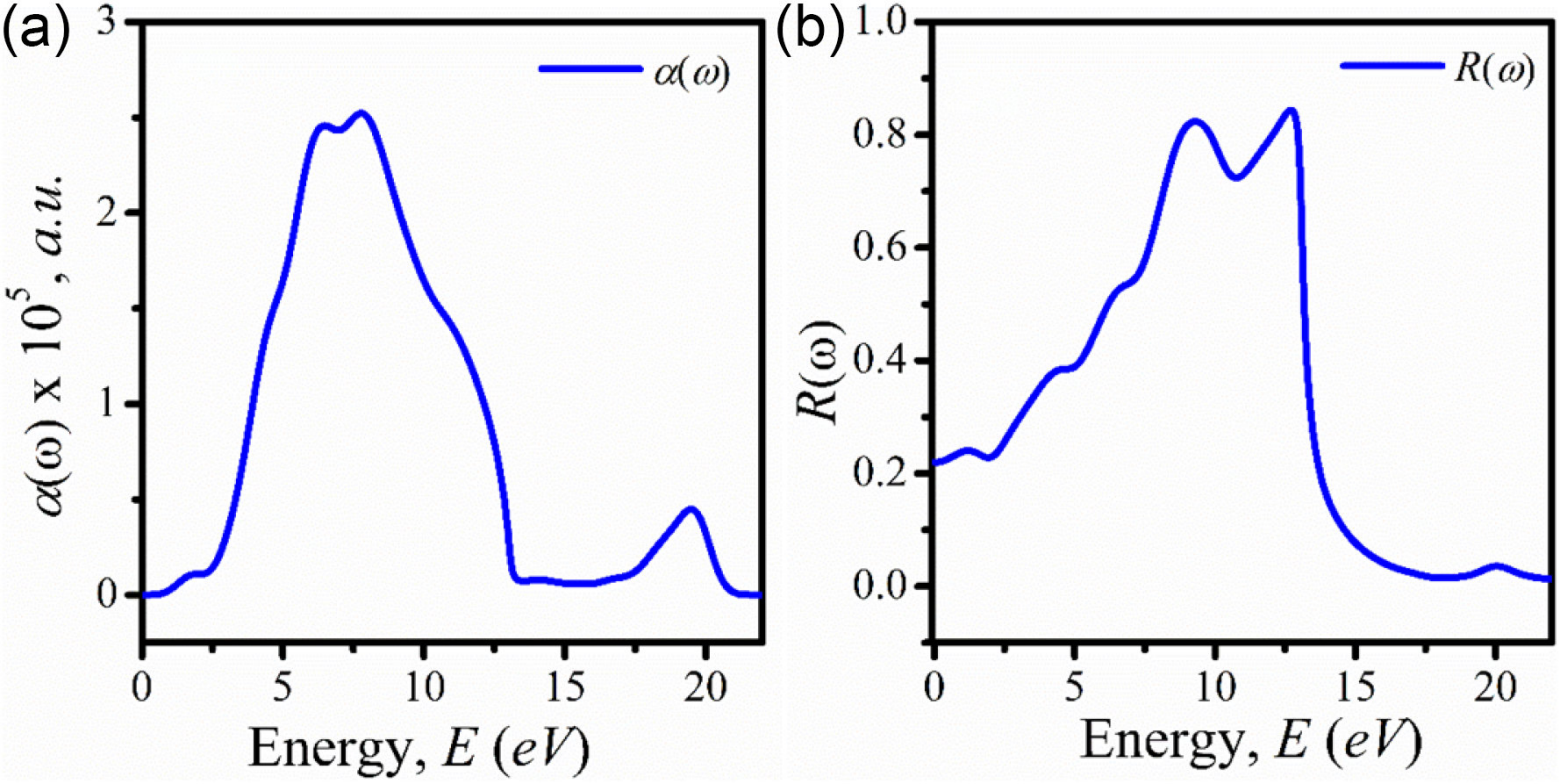
Optical spectra of cubic spinel ZnIn_2_S_4_: (a) absorption coefficient and (b) reflectivity.

The absorption coefficient reaches a maximum value of 8.76 × 10^5 ^cm^−1^ at approximately 7.72 eV, after which it decreases sharply with increasing photon energy. The exceptionally high absorption coefficient indicates that ZnIn_2_S_4_ can absorb a large fraction of incident photons within a very thin surface layer, resulting in efficient generation of photogenerated electron–hole pairs. The strong absorption originates from the high density of available electronic states and the enhanced transition probability associated with the hybridized orbitals participating in interband electronic transitions.

The high value of *α*(*ω*) demonstrates that ZnIn_2_S_4_ possesses excellent light‐harvesting capability over a broad energy range extending from the visible to the ultraviolet region. Such strong optical absorption minimizes photon transmission through the material and maximizes the utilization of incident light, thereby increasing the efficiency of photo‐induced carrier generation. The large number of generated electron–hole pairs can subsequently participate in electrical conduction or surface redox reactions, depending on the application.

The remarkably high absorption coefficient, together with the favorable electronic structure, indicates that ZnIn_2_S_4_ is a promising material for photocatalytic, photoelectrochemical, and photovoltaic applications, where efficient photon absorption is essential for maximizing solar‐energy utilization. In addition, its strong optical absorption makes it attractive for photodetectors, optical absorbers, and other visible‐light‐responsive optoelectronic devices requiring efficient conversion of incident light into electrical or chemical energy.

Reflectivity, *R*(*ω*), represents the fraction of incident electromagnetic radiation reflected from the surface of a material. It provides valuable information about the optical losses occurring at the material surface and determines how much incident light is reflected rather than transmitted or absorbed. Materials with low reflectivity generally exhibit higher light absorption because a larger proportion of the incident photons enters the material and participates in electronic excitation. Therefore, reflectivity is an important parameter for evaluating semiconductors intended for photovoltaic, photocatalytic, and optoelectronic applications. It is calculated from the complex refractive index according to [66, 67]

$$R\left(\omega \right)={\left|\frac{N(\omega )-1}{N(\omega )+1}\right|}^{2}=\frac{{\left(n-1\right)}^{2}+k^{2}}{{\left(n+1\right)}^{2}+k^{2}}$$

where N(ω)=n(ω)+ik(ω) is the complex refractive index, and *n*(*ω*) and *k*(*ω*) are the refractive index and extinction coefficient, respectively.

The calculated reflectivity spectrum of cubic ZnIn_2_S_4_ is presented in Figure 5b. The reflectivity remains relatively low throughout the visible region and gradually increases with photon energy, indicating that most of the incident light can penetrate the material instead of being reflected from its surface. Two prominent reflectivity peaks are observed at approximately 9.2 and 12.7 eV, corresponding to strong electronic excitations in the ultraviolet region. These peaks originate from intense interband electronic transitions involving the hybridized Zn 3*d*/S 3*p* valence‐band states and the In 5*s*/In 5*p* conduction‐band states. The second peak appears close to the second plasma energy (~12.9 eV), indicating enhanced collective electronic response at higher photon energies. Beyond these maxima, the reflectivity decreases sharply, suggesting reduced reflection as the optical response weakens at higher energies.

The relatively low reflectivity over a broad photon‐energy range is a highly desirable characteristic because it minimizes optical losses and allows a larger fraction of incident light to enter the material. The increased photon penetration enhances optical absorption and promotes the generation of photogenerated electron–hole pairs, thereby improving light utilization efficiency. This behavior is fully consistent with the high absorption coefficient obtained for ZnIn_2_S_4_, demonstrating that the material absorbs rather than reflects a significant portion of the incident radiation.

The combination of low reflectivity and strong optical absorption indicates that ZnIn_2_S_4_ is an excellent light‐harvesting semiconductor.

#### 3.4.4 Photoconductivity and Dielectric Loss

Photoconductivity, *σ*(*ω*), describes the increase in the electrical conductivity of a material under photon irradiation due to the generation of electron–hole pairs. When the energy of the incident photons exceeds the bandgap energy, electrons are excited from the valence band to the conduction band, leaving behind holes in the valence band. These photogenerated charge carriers contribute to electrical conduction, making photoconductivity an important parameter for evaluating the photoresponse, charge transport capability, and optoelectronic performance of semiconductor materials. Since photoconductivity depends on the generation, separation, and transport of charge carriers, it provides valuable insight into the efficiency of photoinduced electronic processes.

The optical photoconductivity is directly related to the absorptive component of the dielectric function and is expressed as [66, 72, 73]

$$\sigma \left(\omega \right)=\frac{\omega \varepsilon_{0}\varepsilon_{i}(\omega )}{4\pi }$$

where *ω* is the angular frequency of the incident photon, *ε*_0_ is the permittivity of free space, and *ε*_*i*_(*ω*) is the imaginary part of the dielectric function. Alternatively, the optical conductivity can be evaluated from the absorption coefficient and refractive index according to [74, 75]

$$\sigma \left(\omega \right)=\frac{\alpha nc}{4\pi }$$

where *α* is the absorption coefficient, *n* is the refractive index, and *c* is the speed of light. These relationships indicate that photoconductivity is directly associated with the optical absorption and electronic transitions occurring within the material.

The calculated real and imaginary photoconductivity spectra of cubic ZnIn_2_S_4_ are presented in Figure 6a. The real photoconductivity, *σ*_r_(*ω*), is nearly zero at low photon energies because photons with energies below the optical bandgap cannot excite electrons into the conduction band. As the photon energy exceeds the bandgap energy, *σ*_r_(*ω*) increases rapidly, indicating the onset of efficient photocarrier generation through interband electronic transitions. The real photoconductivity reaches its maximum value at approximately 5.6 eV, corresponding to the highest concentration of photoexcited carriers contributing to electrical conduction. This pronounced peak primarily originates from transitions between the hybridized Zn 3*d*/S 3*p* valence‐band states and the In 5*s*/In 5*p* conduction‐band states, where the transition probability is highest. Beyond this energy, the real photoconductivity decreases gradually because the density of available electronic states and the probability of optical transitions become lower.

**FIGURE 6:**
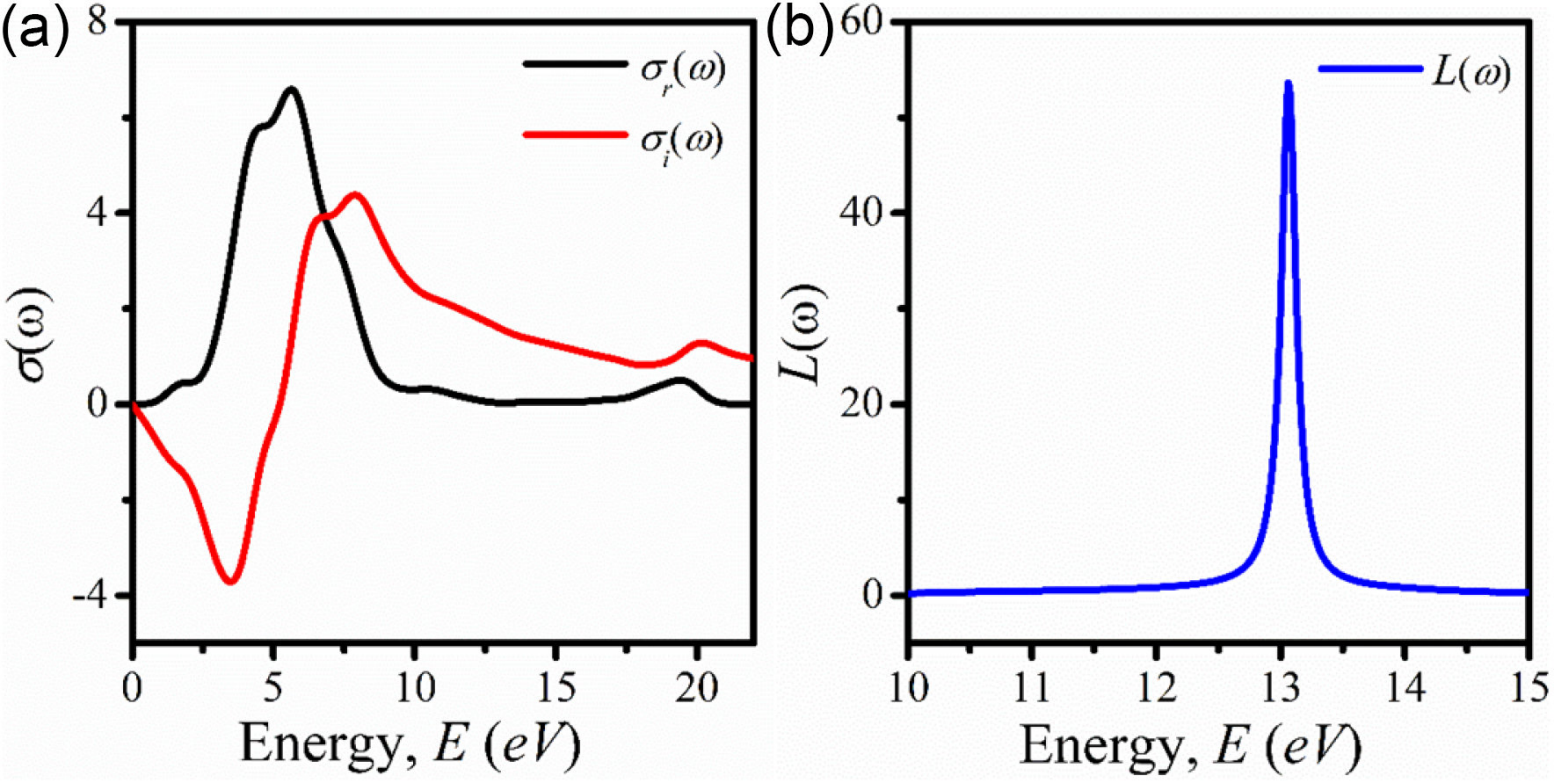
Photoresponse characteristics of cubic spinel ZnIn_2_S_4_: (a) photoconductivity spectra and (b) energy‐loss function.

The imaginary photoconductivity, *σ*_*i*_(*ω*), initially exhibits negative values at low photon energies owing to the phase difference between the induced current and the oscillating electromagnetic field. It is observe that, as the photon energy increases, *σ*_*i*_(*ω*) spectra rises rapidly after the onset of interband transitions and attains its maximum at approximately 7.9 eV. The subsequent decrease at higher photon energies indicates the gradual reduction of the reactive response of photoexcited carriers. The broad photoconductivity spectrum demonstrates that ZnIn_2_S_4_ maintains effective photo‐induced carrier dynamics over a wide energy range extending from the visible to the ultraviolet region [76, 77].

The strong photoconductivity observed above the bandgap energy indicates that ZnIn_2_S_4_ can efficiently generate, separate, and transport photogenerated charge carriers under light irradiation. This observation is consistent with the previously calculated small electron effective mass (0.129*m*_e_) and high electron mobility (136.3 cm^2 ^V^−1 ^s^−1^), which facilitate rapid electron transport and suppress electron‐hole recombination. Consequently, the efficient migration of photogenerated electrons enhances the probability of surface redox reactions, making ZnIn_2_S_4_ particularly attractive for photocatalytic applications.

The dielectric loss function, *L*(*ω*), describes the energy lost by fast‐moving charged particles, such as electrons, as they propagate through a material. It provides valuable information about the collective oscillation of electrons (plasmons) and is widely used to determine the plasma frequency and energy dissipation characteristics of semiconductors. The energy loss function is particularly important for understanding electron‐energy‐loss spectroscopy (EELS), optical excitation processes, and the screening behavior of charge carriers. It is mathematically expressed as [66, 78, 79]

L(ω)=Im −1ε(ω)=εi(ω)εr2(ω)+εi2(ω)

where *ε*_r_(*ω*) and *ε*_*i*_(*ω*) are the real and imaginary parts of the complex dielectric function, respectively. The loss function reaches its maximum when the real dielectric constant approaches zero while the imaginary dielectric constant remains relatively small, corresponding to the plasma resonance of the material.

The calculated dielectric loss spectrum of cubic ZnIn_2_S_4_ is presented in Figure 6b. The loss function remains nearly zero over most of the investigated energy range, indicating negligible energy dissipation by fast electrons at low photon energies. A sharp and intense peak is observed at approximately 13.05 eV, which corresponds to the bulk plasma frequency of ZnIn_2_S_4_. This energy closely agrees with the second plasma peak (~12.90 eV) identified from the dielectric function analysis, confirming the occurrence of strong collective oscillations of the electron cloud at this energy.

The sharp and narrow nature of the loss peak indicates that plasma resonance occurs within a limited energy range and is associated with a significant transfer of energy from incident electrons to the collective electronic oscillations of the material. At the plasma frequency, the real dielectric constant approaches zero, causing the material to exhibit maximum dielectric loss and strong interaction with energetic electrons. Beyond this resonance energy, the loss function decreases rapidly, indicating reduced energy dissipation as the plasma oscillation weakens.

The observed dielectric loss behavior is consistent with the dielectric constant and reflectivity spectra. The pronounced loss peak near 13.05 eV coincides with the high‐energy reflectivity maximum and the plasma resonance identified from the real dielectric function, demonstrating the strong correlation among these optical parameters. The relatively low dielectric loss throughout the visible region indicates that ZnIn_2_S_4_ experiences minimal energy dissipation under visible‐light irradiation, allowing photoexcited charge carriers to utilize the absorbed photon energy more effectively rather than losing it through collective electronic damping.

The calculated optical parameters reveal that cubic ZnIn_2_S_4_ possesses favorable intrinsic optical characteristics for light‐responsive semiconductor applications. The strong dielectric response and refractive index indicate effective interaction with electromagnetic radiation and potential suitability for optical modulation and light‐management applications. The enhanced absorption coefficient, extinction coefficient, and optical conductivity demonstrate efficient photon absorption and photo‐induced carrier excitation, which are important characteristics for photodetectors, photoelectrochemical systems, and photovoltaic‐related applications. In addition, the low reflectivity in the visible region and limited dielectric loss suggest reduced optical losses and improved utilization of incident photons. These combined optical features indicate that cubic ZnIn_2_S_4_ could be considered a potential candidate for next‐generation visible‐light‐responsive optoelectronic and semiconductor devices, although practical device performance requires further experimental evaluation and optimization of device‐level parameters.

### 3.5 Polycrystalline Elastic Properties and Elastic Anisotropy

The elastic properties of crystalline materials describe their response to external mechanical stress and provide essential information about mechanical stability, stiffness, compressibility, ductility, and resistance to deformation. For cubic crystals, the elastic response is completely defined by three independent elastic stiffness constants, namely, *C*_11_, *C*_12_, and *C*_44_, which were calculated using the stress–strain method within the framework of DFT. Here, *C*_11_ represents the resistance against longitudinal deformation, *C*_12_ describes the coupling between different crystallographic directions, and *C*_44_ corresponds to the resistance against shear deformation. The mechanical stability of cubic ZnIn _2_S _4_ was evaluated using the Born stability criteria [80, 81]:

$$C_{11}>0,\;\;C_{44}>0,\;\;C_{11}-C_{12}>0,\;\;C_{11}+2C_{12}>0$$

Satisfaction of these conditions confirms that the crystal structure is mechanically stable against small elastic distortions.

The macroscopic elastic properties of cubic ZnIn _2_S _4_ were obtained from the calculated elastic constants using the VRH approximation. The bulk modulus (*K*), which represents the resistance of a material against uniform volume compression, is expressed as [64, 65, 82]

$$K=\frac{C_{11}+2C_{12}}{3}$$

The shear modulus (*G*), which measures the resistance to shape deformation, was calculated using the Voigt and Reuss approximations. The Voigt shear modulus is given by [65]

$$G_{V}=\frac{C_{11}-C_{12}+3C_{44}}{5}$$

whereas the Reuss shear modulus is expressed as [82]

$$G_{R}=\frac{5(C_{11}-C_{12})C_{44}}{4C_{44}+3(C_{11}-C_{12})}$$

The Hill average provides a reliable estimation of the effective shear modulus for polycrystalline materials and is calculated as [64]

$$G_{H}=\frac{G_{V}+G_{R}}{2}$$

For cubic crystals, the bulk modulus is identical in both Voigt and Reuss approximations; therefore, the Hill bulk modulus is given by

$$K_{H}=\frac{K_{V}+K_{R}}{2}=K$$

The Young’s modulus (*E*), which describes the stiffness of the material against uniaxial deformation, and Poisson’s ratio (ν), which represents the transverse strain response under tensile stress, were calculated using [64]

$$E=\frac{9KG}{3K+G}$$

$$\nu =\frac{3K-2G}{2(3K+G)}$$

The elastic anisotropy of cubic ZnIn _2_S _4_ was evaluated using the Zener anisotropy factor (*A*) [83]:

$$A=\frac{2C_{44}}{C_{11}-C_{12}}$$

where *A *= 1 corresponds to a perfectly elastically isotropic crystal, while deviations from unity indicate directional dependence of the elastic response.

The Pugh ratio *G*/*K* was calculated to evaluate the ductile or brittle nature of ZnIn_2_S_4_, where a lower value indicates ductile behavior, while a higher value suggests a brittle response. Materials with *G*/*K *< 0.57 generally exhibit ductile behavior, whereas those with *G*/*K *> 0.57 are considered brittle. Additionally, Poisson’s ratio provides further insight into bonding characteristics and deformation behavior, where values above approximately 0.26 are commonly associated with ductile materials [84].

To obtain a detailed understanding of directional mechanical behavior, the anisotropic variations of Young’s modulus, shear modulus, Poisson’s ratio, and linear compressibility were analyzed using the ELATE package [85]. These directional properties quantify the dependence of elastic response on crystallographic orientation by comparing the maximum and minimum values along different directions.

The calculated elastic constants were further used to determine the effective mechanical properties of polycrystalline ZnIn _2_S _4_. The Voigt approximation assumes uniform strain throughout the crystal, providing the upper limit of elastic moduli, whereas the Reuss approximation assumes uniform stress and gives the lower limit. The Hill average, obtained from the arithmetic mean of the Voigt and Reuss limits, provides a more realistic estimation of the mechanical properties of polycrystalline materials and is therefore widely adopted for theoretical investigations [64]. The obtained elastic parameters, including bulk modulus, shear modulus, Young’s modulus, Poisson’s ratio, and anisotropy factors, are summarized in Table 3. These parameters provide comprehensive insight into the mechanical stability, deformation resistance, and structural reliability of cubic ZnIn _2_S _4_ for potential functional applications.

The calculated polycrystalline elastic properties of cubic spinel ZnIn_2_S_4_, including the bulk modulus, Young’s modulus, shear modulus, and Poisson’s ratio obtained from the Voigt, Reuss, and Hill approximations, are summarized in Table 3. The corresponding directional dependence of the elastic properties is illustrated through the two‐dimensional and three‐dimensional elastic surface plots in Figures 7 and 8, respectively. These elastic parameters provide essential information about the mechanical stiffness, compressibility, deformation resistance, and bonding characteristics of the material.

**FIGURE 7:**
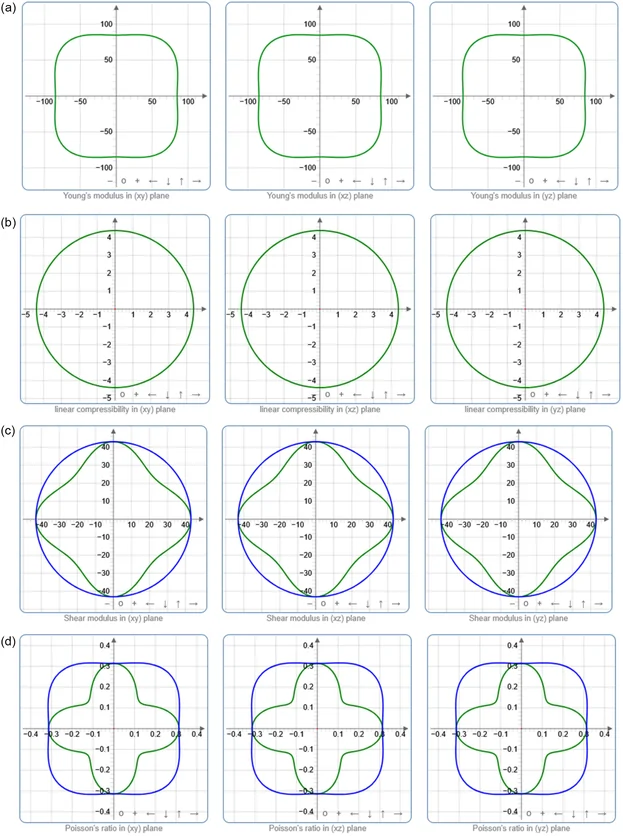
Two‐dimensional directional elastic properties of cubic spinel ZnIn_2_S_4_: (a) Young’s modulus, (b) linear compressibility, (c) shear modulus, and (d) Poisson’s ratio.

**FIGURE 8:**
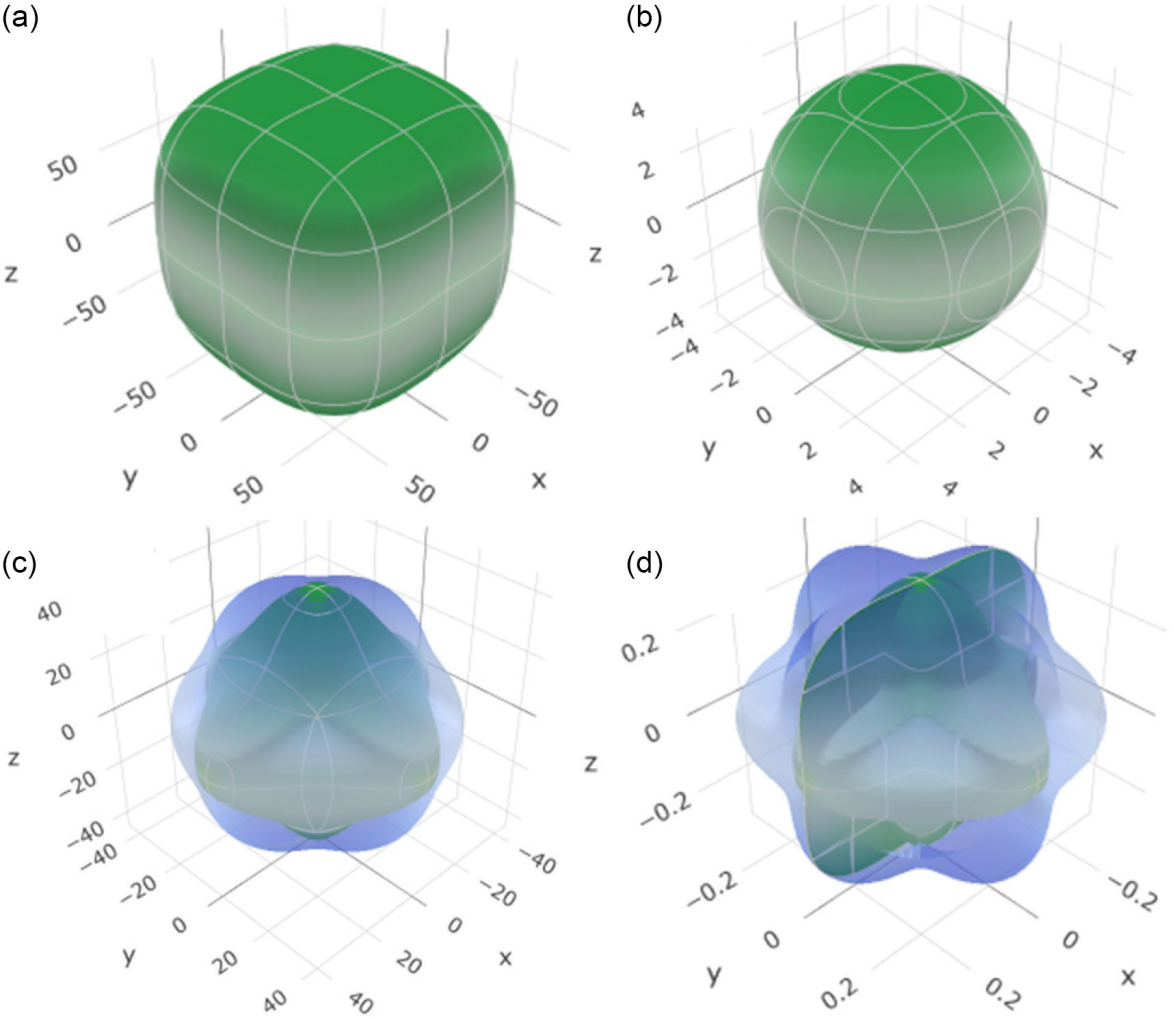
3D directional elastic properties of cubic spinel ZnIn_2_S_4_: (a) Young’s modulus, (b) linear compressibility, (c) shear modulus, and (d) Poisson’s ratio.

The bulk modulus represents the resistance of a material to uniform compression. The calculated Voigt, Reuss, and Hill bulk moduli of cubic ZnIn_2_S_4_ are identical, with a value of 75.896 GPa, indicating consistent elastic behavior. This value is higher than the previously reported cubic ZnIn_2_S_4_ value of 66.04 GPa [56] and the hexagonal ZnIn_2_S_4_ value of 18.58 GPa [53], suggesting stronger resistance to volumetric deformation. This moderate bulk modulus suggests that ZnIn_2_S_4_ possesses reasonable resistance against volume deformation while maintaining structural flexibility. The good agreement between the three approximations confirms the reliability of the calculated elastic response.

The Young’s modulus represents the resistance of a material against uniaxial deformation. The calculated Voigt, Reuss, and Hill Young’s moduli of cubic ZnIn_2_S_4_ are 99.376, 97.692, and 98.535 GPa, respectively, showing consistent elastic behavior. The calculated Voigt value is higher than the reported values for cubic ZnIn_2_S_4_, 78.75 GPa [56] and hexagonal ZnIn_2_S_4_, 21.73 GPa [53]. The relatively high Young’s modulus demonstrates that the material possesses considerable stiffness and can withstand external mechanical stress without significant deformation. The two‐dimensional and three‐dimensional Young’s modulus surfaces shown in Figures 7a and 8a reveal a moderate directional dependence, where the maximum and minimum Young’s moduli are found to be 108.70 and 84.81 GPa, respectively. The calculated Young’s modulus anisotropy factor (AE = 1.282) indicates slight elastic anisotropy, meaning that the mechanical stiffness varies moderately with crystallographic direction.

The shear modulus describes the resistance of a material against shape deformation under shear stress. The calculated Voigt, Reuss, and Hill shear moduli are 38.765, 37.998, and 38.382 GPa, respectively. The Voigt shear modulus is higher than the reported values for cubic ZnIn_2_S_4_, 31.97 GPa [56], and hexagonal ZnIn_2_S_4_, 8.33 GPa [53], indicating stronger resistance to shear deformation. The relatively lower magnitude of the shear modulus compared with the bulk modulus indicates that ZnIn_2_S_4_ is more resistant to volume compression than shear deformation. The directional variation of the shear modulus is presented in Figures 7c and 8c. The minimum and maximum shear moduli are calculated to be 32.278 and 43.090 GPa, respectively, with a shear anisotropy factor of AG = 1.335. This moderate deviation from unity confirms that the shear response of ZnIn_2_S_4_ depends slightly on the crystallographic direction.

The Poisson’s ratio provides information about the lateral deformation behavior and bonding characteristics of the material. The calculated Poisson’s ratios are 0.28177, 0.28547, and 0.28362 for the Voigt, Reuss, and Hill approximations, respectively. The Hill average value of 0.28362 indicates a stable elastic response and suggests that ZnIn_2_S_4_ exhibits predominantly central‐force ionic bonding characteristics. The directional distribution of Poisson’s ratio shown in Figures 7d and 8d reveals values ranging from 0.17832 to 0.37568, resulting in a Poisson’s ratio anisotropy factor of 2.1068. This larger anisotropy value indicates that the transverse deformation response varies more strongly with crystallographic orientation compared with the variation observed for Young’s modulus and shear modulus.

The compressibility behavior of ZnIn_2_S_4_ was further analyzed through the linear compressibility surfaces, as shown in Figures 7b and 8b. The minimum and maximum linear compressibilities are both calculated as 4.392 TPa^−1^, resulting in a linear compressibility anisotropy factor of *A* *_β_* = 1.0000A. This indicates completely isotropic compressibility, meaning that ZnIn_2_S_4_ exhibits identical resistance to compression along different crystallographic directions. Such behavior is consistent with the high cubic symmetry of the spinel structure.

The elastic stability and mechanical behavior of ZnIn_2_S_4_ were further examined through the eigenvalues of the stiffness matrix, which are listed in Table 3. The six eigenvalues are calculated as 43.090, 43.090, 43.090, 64.555, 64.555, and 227.690 GPa. All eigenvalues are positive, confirming that the stiffness matrix is positive definite and that the optimized cubic structure satisfies the mechanical stability requirements. The largest eigenvalue corresponds to the strongest elastic response of the crystal against deformation, whereas the smaller eigenvalues represent softer deformation modes associated with shear and transverse distortions.

The calculated elastic parameters, hardness, fracture toughness, and ductility characteristics provide valuable information about the mechanical reliability of ZnIn_2_S_4_ for practical applications. The mechanical stability and favorable deformation resistance indicate that the material can maintain structural integrity under external mechanical stress, making it suitable for functional applications where durability is required. The obtained mechanical properties are particularly important for semiconductor devices, thin‐film systems, photovoltaic structures, and photoelectrochemical components, where materials may experience thermal expansion, fabrication‐induced stress, and operational loading. Therefore, the comprehensive mechanical analysis confirms that ZnIn_2_S_4_ possesses sufficient structural stability and mechanical robustness for potential use in advanced electronic, optoelectronic, and renewable‐energy technologies.

### 3.6 Phonon Dispersion and Dynamical Stability of Cubic ZnIn_2_S_4_

The dynamical stability of cubic ZnIn_2_S_4_ was investigated through phonon dispersion analysis along the high‐symmetry directions of the Brillouin zone, as presented in Figure 9. Phonon frequencies were obtained by solving the dynamical matrix equation:

$$D_{ij}^{\alpha \beta }\left(q\right)e_{j}^{\beta }\left(q\right)=\omega^{2}e_{j}^{\alpha }(q)$$

**FIGURE 9:**
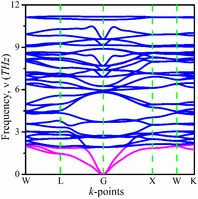
Calculated phonon dispersion spectrum of cubic ZnIn_2_S_4_ along the W–L–G–X–W–K high‐symmetry path. The absence of imaginary phonon modes confirms the dynamical stability of the optimized structure.

where *D*(*q*) represents the dynamical matrix, *e*(*q*) is the phonon eigenvector, and *ω*(*q*) is the phonon frequency corresponding to the wave vector *q* [86, 87]. The presence of only real positive phonon frequencies throughout the Brillouin zone indicates that the crystal structure is dynamically stable, whereas imaginary frequencies represent unstable vibrational modes associated with structural distortions [87, 88].

The primitive cell of cubic ZnIn_2_S_4_ contains 14 atoms, resulting in 42 phonon branches, including three acoustic modes and 39 optical modes. As shown in Figure 9, the phonon spectrum of cubic ZnIn_2_S_4_ exhibits no imaginary frequencies along the W–L–G–X–W–K symmetry directions, confirming the dynamical stability of the optimized structure. The three acoustic branches originate from zero frequency at the G point due to translational invariance and consist of two transverse acoustic (TA) and one longitudinal acoustic (LA) modes. The remaining 39 optical branches arise from relative vibrations between Zn, In, and S atoms. The lower frequency optical modes are mainly associated with vibrations of the heavier Zn and In atoms, whereas the higher frequency modes are dominated by the lighter S atoms. In polar semiconductor systems, these optical modes can be classified into transverse optical (TO) and longitudinal optical (LO) modes, where LO modes generally exhibit higher frequencies than TO modes due to long‐range Coulomb interactions [81, 88]. The absence of negative‐frequency phonon  modes and the stable separation between.

The absence of imaginary phonon modes, together with the positive elastic stiffness eigenvalues, confirms that cubic ZnIn_2_S_4_ satisfies both mechanical and dynamical stability criteria. These results indicate that the optimized structure possesses a robust lattice framework capable of maintaining stability against both static deformation and dynamic atomic vibrations [81].

## 4 Conclusions

In this work, a comprehensive first‐principles investigation was performed to explore the intrinsic structural, electronic, carrier transport, optical, mechanical, and dynamical characteristics of cubic spinel ZnIn_2_S_4_. The optimized structural parameters demonstrate the reliability of the computational approach, where the calculated lattice parameter of 7.555 Å shows excellent agreement with the reported value, with a deviation of only 0.605%. The optimized crystal structure preserves the characteristic cubic spinel framework, confirming the suitability of the relaxed structure for evaluating the fundamental properties of ZnIn_2_S_4_.

The electronic structure analysis reveals that cubic ZnIn_2_S_4_ is an indirect bandgap semiconductor with calculated bandgaps of 1.289 and 1.123 eV using spin‐polarized and nonspin‐polarized GGA‐PBE approaches, respectively. The DOS analysis indicates that the valence‐ and conduction‐band characteristics mainly originate from Zn 3*d*/S 3*p* and In 5*s*/In 5*p* orbital interactions, respectively. These electronic features provide the fundamental basis for understanding the optical excitation and charge transport behavior of the material.

The carrier transport investigation demonstrates favorable electron transport characteristics in cubic ZnIn_2_S_4_. The calculated electron effective mass of 0.129*m*_e_ is significantly lower than the hole effective mass magnitude of 1.970*m*_e_, resulting in estimated electron and hole mobilities of 136.3 and 8.91 cm^2 ^V^−1 ^s^−1^, respectively. These results indicate a strong asymmetry between electron and hole transport, with electron conduction being the dominant contribution to carrier mobility within the crystal.

The optical calculations reveal pronounced interaction between cubic ZnIn_2_S_4_ and electromagnetic radiation. The material exhibits a static dielectric constant of 7.66, a maximum refractive index of 3.34, strong absorption extending from the visible to ultraviolet region, and enhanced photoconductivity response. The low reflectivity in the visible range and broad optical response indicate effective photon utilization and photoinduced charge generation capability.

The mechanical analysis confirms the structural robustness of cubic ZnIn_2_S_4_. The calculated Hill‐averaged bulk modulus, Young’s modulus, shear modulus, and Poisson’s ratio are 75.896 GPa, 98.535 GPa, 38.382 GPa, and 0.28362, respectively. The calculated elastic anisotropy factors reveal moderate directional dependence of mechanical properties, while the positive eigenvalues of the stiffness matrix confirm mechanical stability. In addition, phonon dispersion calculations show the absence of imaginary frequencies throughout the Brillouin zone, demonstrating the dynamical stability of the cubic spinel phase.

Overall, the combined structural stability, suitable semiconducting bandgap, efficient carrier transport, strong optical response, and favorable mechanical characteristics demonstrate that cubic ZnIn_2_S_4_ possesses promising intrinsic properties for semiconductor‐based technologies. These findings indicate that cubic ZnIn_2_S_4_ may serve as a potential material for next‐generation photocatalytic, photovoltaic‐related, photodetection, and other light‐responsive optoelectronic applications, while practical implementation will depend on factors such as defect engineering, interface optimization, and experimental validation.

## Author Contributions

**M. Rahman:** writing – review and editing, writing – original draft, validation, supervision, software, methodology, investigation, formal analysis, conceptualization. **N. Y. Tanisa** and **Y. Yildirim:** writing – review and editing, formal analysis. **M. M. Roshid:** writing – software, review, and editing. **M. S. A. Khan:** writing – review and editing, formal analysis.

## Funding

The authors have nothing to report.

## Conflicts of Interest

The authors declare no conflicts of interest.

## Data Availability

Relevant data from this study are available from the corresponding author upon a reasonable request.
